# Controlled Release Urea as a Nitrogen Source for Spring Wheat in Western Canada: Yield, Grain N Content, and N Use Efficiency

**DOI:** 10.1100/tsw.2001.309

**Published:** 2001-10-30

**Authors:** Lenz Haderlein, T.L. Jensen, R.E. Dowbenko, A.D. Blaylock

**Affiliations:** Agrium, Bag 20, Redwater, AB T0A 2W0, USA; Agrium, 13131 Lake Fraser Drive SE, Calgary, AB T2J 7E8, USA; Agrium US, 4582 S. Ulster St., Suite 1700, Denver, CO 80237, USA

**Keywords:** urea, coated, denitrification, wheat, nitrous oxide

## Abstract

Controlled release nitrogen (N) fertilizers have been commonly used in horticultural applications such as turf grasses and container-grown woody perennials. Agrium, a major N manufacturer in North and South America, is developing a low-cost controlled release urea (CRU) product for use in field crops such as grain corn, canola, wheat, and other small grain cereals. From 1998 to 2000, 11 field trials were conducted across western Canada to determine if seed-placed CRU could maintain crop yields and increase grain N and N use efficiency when compared to the practice of side-banding of urea N fertilizer. CRU was designed to release timely and adequate, but not excessive, amounts of N to the crop. Crop uptake of N from seed-placed CRU was sufficient to provide yields similar to those of side-banded urea N. Grain N concentrations of the CRU treatments were higher, on average, than those from side-banded urea, resulting in 4.2% higher N use efficiency across the entire N application range from 25 to 100 kg ha^-1^. Higher levels of removal of N in grain from CRU compared to side-banded urea can result in less residual N remaining in the soil, and limit the possibility of N losses due to denitrification and leaching.

## INTRODUCTION

Inefficient fertilizer use may contribute to environmental degradation, particularly in intensive agricultural systems where the recovery or use efficiency of nutrients by crops is relatively low. For example, it is estimated that nitrogen (N) use efficiency for cereal production worldwide is only 33%[1]. A portion of the N not used by the crop is presumed to be lost to the environment through denitrification, runoff, volatilization, leaching, and gaseous plant emissions. Such losses raise concerns about surface and groundwater contamination and greenhouse gas emissions. Additionally, low use efficiency of nutrients applied as fertilizers results in producers receiving lower economic returns from their investment in fertilizer inputs.

Controlled release fertilizers (CRF) are fertilizers designed to slowly release nutrients at a rate that matches the demand of the crop plants. Such products can be used to maximize fertilizer use efficiency and minimize potential losses to the environment. Increased nutrient-use efficiency may also increase yield and quality of crops, thus providing an economic benefit for growers. Controlled or slow release fertilizers can generally be classified into three types: inorganic compounds of low solubility, low solubility organic N compounds, and coated water-soluble fertilizers. The first two categories have limited potential for agricultural use because their rate of nutrient release is difficult to predict and depends upon factors such as soil type, moisture content, microbial activity, and history of previous usage. Development of polymer-coated fertilizers looks promising for future widespread use in agriculture, since they can be designed to release nutrients in a manner closely matching crop demand. The polymers used are generally very durable and exhibit consistent release rates that are predictable when average temperatures and moisture conditions can be accurately predicted. The rate of nutrient release can be increased or decreased by manipulating properties of the polymer coating.

At present, the use of CRF in agriculture is very limited, accounting for less than 1% of worldwide fertilizer consumption. The main reason for this is cost; CRF can cost between three and eight times as much as a corresponding standard fertilizer. Current usage of CRF is limited primarily to nonagricultural markets such as turf grass. The exception to this trend is Japan, where CRF are widely used on agricultural crops such as rice and vegetables[2].

There is enormous potential for the increased use of CRF in agriculture in North America and Europe if the cost of CRF production can be reduced and advantages such as increased nutrient recovery, improved crop yield and quality, and reduced environmental impacts can be consistently demonstrated. CRF will be adopted most rapidly in locations where N losses are large, in crops where in-season N applications are common, and in crops with shallow rooting systems.

There have been a limited number of published studies that investigate the value of CRF on large acreage agricultural crops. Those that exist generally indicate there is significant value in using CRF under most conditions. For example, N fertilizer application rates on cotton may be reduced by 40% if controlled release rather than conventional fertilizers are used[3]. Trials using polymer-coated urea on winter wheat indicated that there was a 20% yield increase compared to growers' standard practice; research on potatoes, onions, and garlic has also shown a general increase in yield and quality when using CRF[4]. In western Canada, fall application of polymer-coated urea on barley resulted in decreased nitrate accumulation and fertilizer N loss, while spring application of polymer-coated urea resulted in increased crop N uptake[5].

The objective of this study was to determine whether or not a controlled release N source could be used beneficially in the northern Great Plains. A common practice used in this region consists of placing the fertilizer in a concentrated band below the soil surface prior to crop seeding. Many growers have also moved toward conservation tillage practices to conserve moisture, reduce soil erosion, and reduce fuel and labor inputs[6]. The combination of these practices with cool soils and limited rainfall have already increased N use efficiency, compared to earlier broadcast N fertilizer followed by intense tillage operations to incorporate the fertilizer. A suitable controlled-release N fertilizer for this region would have an N release pattern that matches crop uptake, and must be able to increase N use efficiency even further. The implications for this are that less residual N is left susceptible to over-winter losses and that application rates could potentially be reduced while maintaining crop yields.

## MATERIALS AND METHODS

From 1998 to 2000, 11 field trials were established at sites in Alberta and Saskatchewan to measure yield response of wheat to conventional and controlled release urea (CRU) N fertilizer (Table 1). The experiments were established as randomized complete blocks with four replicates. The three N fertilizer source and placement combinations were urea side-banded 30 mm beside and 30 mm below the seed row, CRU applied in the seed row, and seed-row placed regular urea. Four different rates of N were applied for each application method, those being 25, 50, 75, and 100 kg N $ha^{-1}$. A zero-N treatment was also included. The CRU product used has a polymer coating with properties that allow the release of all available N over time. In laboratory tests the entire N was solubilized within 40 to 50 days when immersed in water at 23°C (Fig. 1). Sites were selected from a variety of soil and climatic regions in Alberta and Saskatchewan with low to moderate soil test N levels (<15 mg $NO_3$-N $kg^{-1}$ in the upper 0.3 m of soil). Soil pH levels were neutral to acid at all sites, so severe seedling ammonia toxicity from side-banded urea would not be expected[7]. Spring wheat (*Triticum aestivum* L. cvs. CDC Teal [1998] and AC Barrie [1999-2000]) was seeded directly into standing stubble using a research air seeder equipped with commercially available Flexi-Coil Stealth™ seed openers on a 0.2-m spacing, with 38-mm spread of seed and fertilizer. At one site (Ellerslie in 1999), excessive moisture early in the growing season delayed seeding, and barley (*Hordeum vulgare* L. cv. Manley) with a shorter required growing season was substituted. Phosphate and potassium fertilizers (mono-ammonium phosphate [12-51-0] and KCl [0-0-60]) were seed-placed at 25 kg $P_2O_5$ $ha^{-1}$ and 25 kg $K_2O$ $ha^{-1}$. Wheat and barley were seeded to achieve a target stand of 150 plants $m^{-2}$. Weed control consisted of a pre-emergent application of a nonselective herbicide followed by application of registered post-emergent selective herbicide.

Plant stand densities were determined, counting the number of seedlings in two random 1-m sections of row, from within each plot. These measurements were performed approximately 30 days after seeding at each site. Grain yields were determined by threshing the entire plot using a plot combine, followed by determination of grain weight and moisture. For grain N analysis, a subsample of grain from each plot was ground to pass a 2-mm sieve and analyzed using a combustion analyzer[8]. Data were analyzed using a general linear model procedure[9]. For combined site analysis, the site was considered as a random variable. Treatment differences and response curves were determined by using preplanned orthogonal contrasts.

## RESULTS AND DISCUSSION

### Plant Stand Density

Uncoated urea placed in the seed row significantly reduced plant stand densities compared to the control at 8 of 11 sites (Table 2). In all cases, plant stands with seed-placed CRU were not significantly different from side-banded urea, even under conditions in which plant establishment was negatively affected by placing uncoated urea in the seed row. CRU placement in the seed row would allow growers to place all their fertilizer with the seed without adding any of the expense or inconvenience of using side-banding attachments. Side-banding equipment is presently used to provide a margin of safety from ammonia toxicity when urea hydrolyzes in the soil. Stand reduction from seed-placed urea was more severe at higher N rates, but N rate did not affect plant establishment for the side-banded urea or CRU treatments. Seed-placed CRU was as effective as side-banding at creating a physical barrier between urea N fertilizer and germinating seedlings.

**TABLE 1**
**Sites, Seeding, and Harvest Dates of the Experiments**

| Site | Soil pH (0 – 0.3 m) | Soil Test N (mg kg$^{-1}$ $NO_3$-N) (0 – 0.3 m) | Seeding Date | Harvest Date |
|---|---|---|---|---|
| Battleford, SK – 1998 | 7.0 | 7 | May 13 | Sept. 14 |
| Hussar, AB – 1998 | 6.8 | 8 | May 8 | Sept. 1 |
| Battleford, SK – 1999 | 7.2 | 7 | May 9 | Sept. 8 |
| Athabasca, AB – 1999 | 5.8 | 12 | May 25 | Sept. 20 |
| Ellerslie, AB – 1999 | 6.5 | 7 | June 1 | Sept. 29 |
| Humboldt, SK – 1999 | 7.8 | 5 | May 16 | Sept. 15 |
| Ellerslie, AB – 2000 | 6.7 | 16 | May 2 | Sept. 6 |
| Humboldt, SK – 2000 | 7.8 | 5 | May 8 | Sept. 13 |
| Lloydminster, AB – 2000 | 7.1 | 15 | May 5 | Sept. 11 |
| Ponoka, AB – 2000 | 7.1 | 5 | May 4 | Sept. 20 |
| Wilkie, SK – 2000 | 6.1 | 33 | May 7 | Sept. 11 |

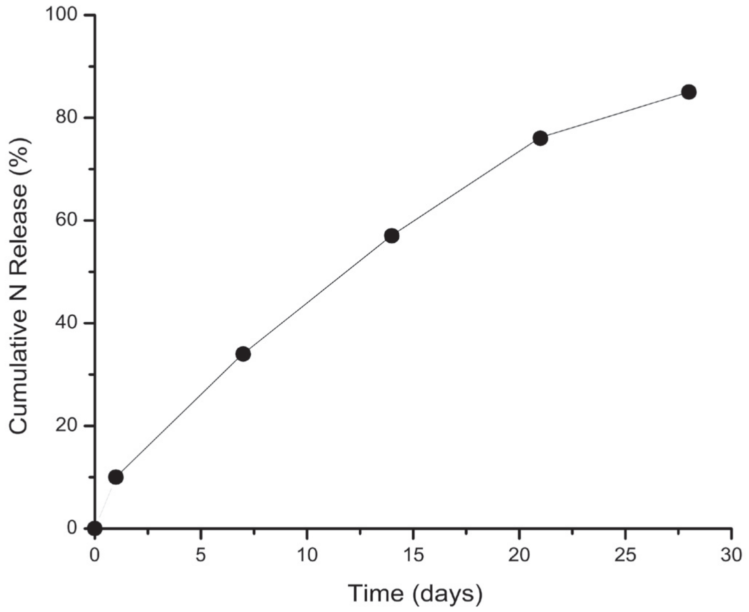

**FIGURE 1.** N release from CRU in water at 23°C.

## Grain Yield

Yield results varied widely in these experiments. Barley at Ellerslie in 1999 had the highest yields (Table 3), while wheat at Ponoka in 2000 had the lowest yields as a result of hail damage. The range in yields is reflective of the wide variety of soil and growing conditions in the northern Great Plains region.

Added N increased yields at all sites except Athabasca in 1999. Ammonia toxicity from seed-placed conventional urea caused severe damage to plant stands (Table 2) and severe reductions in grain yield, especially at high rates of N (data not presented). Side-banded urea and seed-placed CRU yields were not significantly different in any of the experiments. When data from the sites were combined, the yield response to increasing N

**TABLE 2**
**Means of Plant Stand at 11 Sites from 1998 to 2000**

| Site | Plants $m^{-2}$ | | | | Model Effect | | | Contrasts | | |
|---|---|---|---|---|---|---|---|---|---|---|
| | No Added N | Seed-Placed Urea | Side-Banded Urea | Seed-Placed CRU | N Source | N Rate | N Source × N Rate | Seed-Placed Urea vs. No N | (Side-Banded Urea and Seed-Placed CRU) vs. No N | Side-Banded Urea vs. Seed-Placed CRU |
| Battleford – 1998 | 63 | 48 | 70 | 65 | ** | NS | NS | ** | NS | NS |
| Hussar – 1998 | 96 | 50 | 96 | 81 | ** | ** | ** | ** | NS | NS |
| Battleford – 1999 | 129 | 131 | 129 | 124 | NS | NS | NS | NS | NS | NS |
| Athabasca – 1999 | 184 | 124 | 171 | 146 | ** | ** | NS | ** | ** | NS |
| Ellerslie – 1999 | 135 | 129 | 124 | 149 | ** | NS | NS | ** | NS | NS |
| Humboldt – 1999 | 190 | 203 | 215 | 199 | NS | NS | NS | NS | NS | NS |
| Ellerslie – 2000 | 115 | 108 | 107 | 119 | NS | NS | NS | NS | NS | NS |
| Humboldt – 2000 | 115 | 95 | 118 | 108 | ** | NS | NS | ** | NS | NS |
| Lloydminster – 2000 | 114 | 114 | 100 | 101 | NS | NS | NS | NS | NS | NS |
| Ponoka – 2000 | 52 | 102 | 59 | 52 | ** | NS | NS | ** | NS | NS |
| Wilkie – 2000 | 61 | 53 | 50 | 32 | ** | ** | NS | ** | ** | NS |

*Note*: Treatment means and contrasts were performed across all N rates from 25 to 100 kg of applied N $ha^{-1}$.
NS – Model effects and orthogonal contrasts not significantly different.
** – Model effects and orthogonal contrasts significant at $p = 0.05$.

**TABLE 3**
**Means of Grain Yield at 11 Sites from 1998 to 2000**

| Site | Grain Yield (kg $ha^{-1}$) | | | Model Effect | | | Contrasts | | |
|---|---|---|---|---|---|---|---|---|---|
| | No Added N | Side-Banded Urea | Seed-Placed CRU | N Source | N Rate | N Source × N Rate | No N vs. Side-Banded Urea | No N vs. Seed-Placed CRU | Side-Banded Urea vs. Seed-Placed CRU |
| Battleford – 1998 | 908 | 1155 | 1219 | NS | ** | NS | ** | ** | NS |
| Hussar – 1998 | 1605 | 2277 | 2102 | NS | ** | NS | ** | ** | NS |
| Battleford – 1999 | 1125 | 2270 | 2356 | NS | ** | NS | ** | ** | NS |
| Athabasca – 1999 | 1266 | 1176 | 1291 | NS | NS | NS | NS | NS | NS |
| Ellerslie – 1999 | 5296 | 5962 | 6198 | NS | ** | NS | ** | ** | NS |
| Humboldt – 1999 | 1329 | 2537 | 2431 | NS | ** | NS | ** | ** | NS |
| Ellerslie – 2000 | 4523 | 5010 | 4930 | NS | ** | NS | ** | ** | NS |
| Humboldt – 2000 | 1345 | 2531 | 2350 | NS | ** | NS | ** | ** | NS |
| Lloydminster – 2000 | 805 | 1270 | 1303 | NS | ** | NS | ** | ** | NS |
| Ponoka – 2000 | 717 | 1112 | 1114 | NS | ** | NS | ** | ** | NS |
| Wilkie – 2000 | 1200 | 1745 | 1634 | NS | ** | NS | ** | ** | NS |

*Note:* Treatment means and contrasts were performed across all N rates from 25 to 100 kg of applied N $ha^{-1}$.
NS – Model effects and orthogonal contrasts not significantly different.
** – Model effects and orthogonal contrasts significant at $p = 0.05$.

was determined by contrast to be a quadratic function (Fig. 2). There was no significant difference between N sources in the combined data. This suggests that seed-row applied CRU is a viable alternative to side-banded urea and does not result in significant yield losses, despite its close proximity to the seed. The lack of a yield advantage with CRU may be attributed to several factors. First, N applied in a band at the time of seeding is an efficient N-placement method in this region, resulting in higher

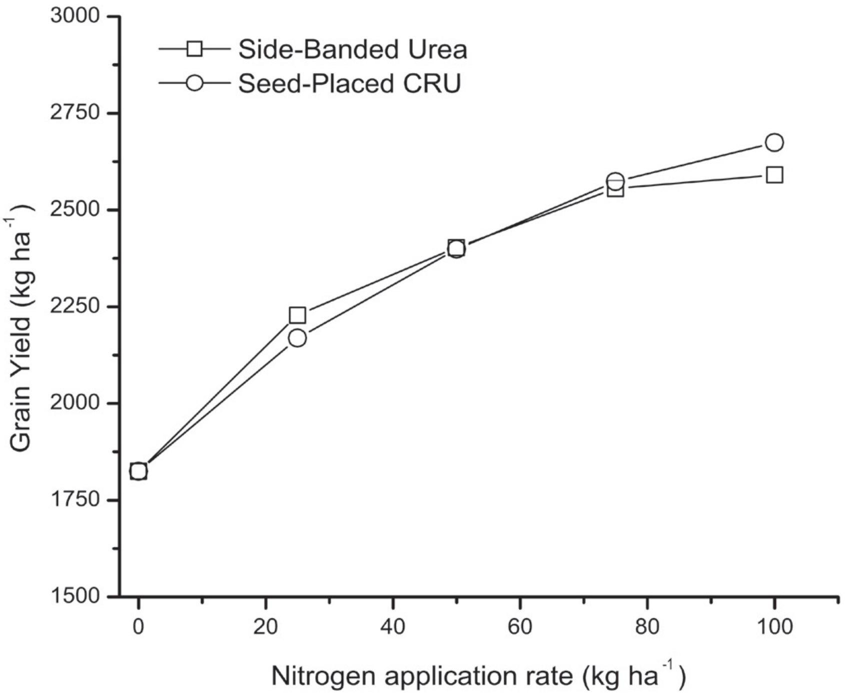

**FIGURE 2.** Yield response of wheat and barley experiments (means of 11 experiments) to added N sources. Yield responses determined to be quadratic by contrast at $p = 0.05$.

yields than any of the other accepted practices such as broadcasting or fall banding[10]. Second, moisture availability generally sets an upper limit of what yields can be attained with application of good N management. Lastly, N losses in this environment are relatively low compared to other areas of the world because of the semiarid climate and cold winters. Large yield differences between an unprotected N source (urea) and CRU are not expected unless significant N losses from leaching or denitrification occur during the growing season. We did not observe conditions conducive to large N losses from leaching and denitrification, nor were measurements of leaching or denitrification conducted.

## Grain N Content

Grain N content in applied-N treatments was greater than the N content of the no-N treatments at 8 of 11 sites (Table 4). Grain N in CRU treatments was significantly greater than banded N treatments at Hussar and Humboldt in 2000.

There was not a significant difference in grain N contents between seed-row CRU and side-banded urea at 9 out of 11 sites. However, when the data from all experiments were combined, there was an overall difference in grain N between the side-banded urea treatments and the seed-placed CRU treatments (by contrast at $p = 0.05$) (Fig. 3). Grain N content was higher for seed-placed CRU than side-banded urea at the same N rate across the N application range. The release characteristics of CRU allowed more N to be taken up by the plant at later growth stages, when N is likely to be concentrated in the grain. Even though the same rates of N were applied, and yields were similar between the treatments, the amount of N exposed to potential losses from leaching and denitrification was reduced because more of the N applied as fertilizer ended up in the grain of the crop.

A further benefit is that growers in western Canada can receive financial premiums if the wheat they sell has a higher protein content[11]. The growers therefore have a financial motivation to improve the amount of N in the grain of the wheat they produce, even if yields are similar between N sources.

## Nitrogen Removal and Efficiency

In order to further understand the implications of CRU for N management, N recovery was calculated by subtracting mean grain N removal of the no-N check plots in the experiment from grain N removal in treatments and dividing by the applied N rate. The data are expressed as the fraction of applied N that was removed from the field in the grain across all experiments (Fig. 4).

Contrasts of the N recovery functions for the treatments reveal that these functions are decreasing linear functions that are parallel. One would expect that the N recovery in grain would be greater at lower rates of N application, since this is where there is the greatest yield and grain N response per unit of applied N. As N application rate increases, efficiency decreases somewhat. In these experiments, the N use efficiency of seed-placed CRU treatments was higher than that of side-banded urea at all N application rates (Fig. 4). Seed-placed CRU was effective in increasing the N use efficiency by concentrating more N into the grain, and thereby leaving less N in the field to be exposed to

**TABLE 4**
**Means of Grain N Content at 11 Sites from 1998 to 2000**

| | Grain N Content (mg N g$^{-1}$) | | | Model | | | Contrasts | | |
|---|---|---|---|---|---|---|---|---|---|
| Site | No Added N | Side-Banded Urea | Side-Placed CRU | N Source | N Rate | N Source × N Rate | No N vs. Side-Banded Urea | No N vs. Seed-Placed CRU | Side-Banded Urea vs. Seed-Placed CRU |
| Battleford – 1998 | 22.4 | 26.9 | 26.5 | NS | ** | NS | ** | ** | NS |
| Hussar – 1998 | 26.0 | 24.7 | 26.7 | ** | ** | NS | ** | ** | ** |
| Battleford – 1999 | 24.5 | 23.7 | 24.1 | NS | NS | NS | NS | NS | NS |
| Athabasca – 1999 | 27.9 | 30.9 | 30.7 | NS | ** | NS | ** | ** | NS |
| Ellerslie – 1999 | 15.1 | 16.7 | 16.5 | NS | ** | NS | ** | ** | NS |
| Humboldt – 1999 | 23.7 | 24.0 | 24.3 | NS | NS | NS | NS | NS | NS |
| Ellerslie – 2000 | 23.9 | 26.1 | 26.1 | NS | ** | NS | ** | ** | NS |
| Humboldt – 2000 | 22.1 | 22.7 | 32.2 | ** | NS | NS | NS | ** | ** |
| Lloydminster – 2000 | 25.6 | 28.1 | 28.3 | NS | ** | NS | ** | ** | NS |
| Ponoka – 2000 | 31.6 | 32.1 | 32.4 | NS | NS | NS | NS | NS | NS |
| Wilkie – 2000 | 25.4 | 27.1 | 27.5 | ** | NS | NS | ** | ** | NS |

Note: Treatment means and contrasts were performed across all N rates from 25 to 100 kg of applied N ha$^{-1}$.

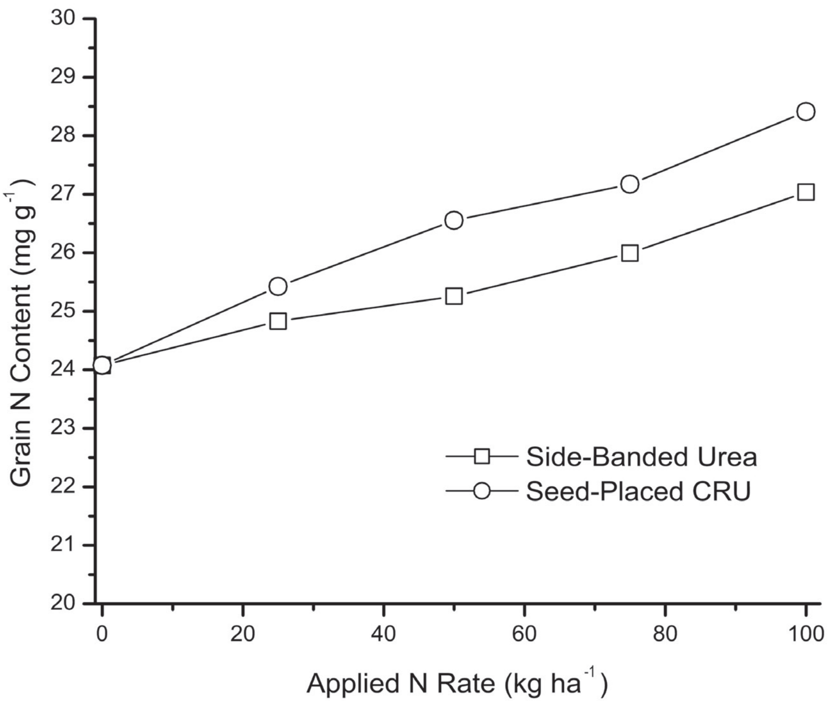

**FIGURE 3.** Grain N response of wheat and barley experiments (means across 11 experiments) to added N sources. Grain N responses to added N are linear by contrast at $p = 0.05$.

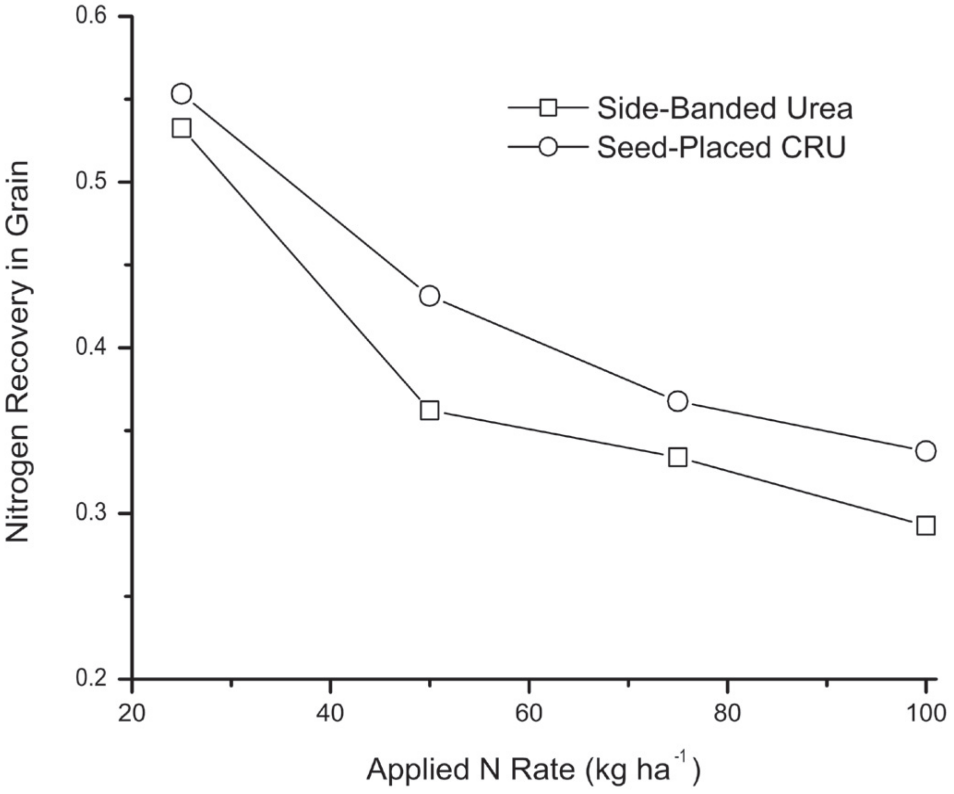

**FIGURE 4.** N recovery in grain from side-banded urea and seed-placed CRU treatments (means across 11 experiments).

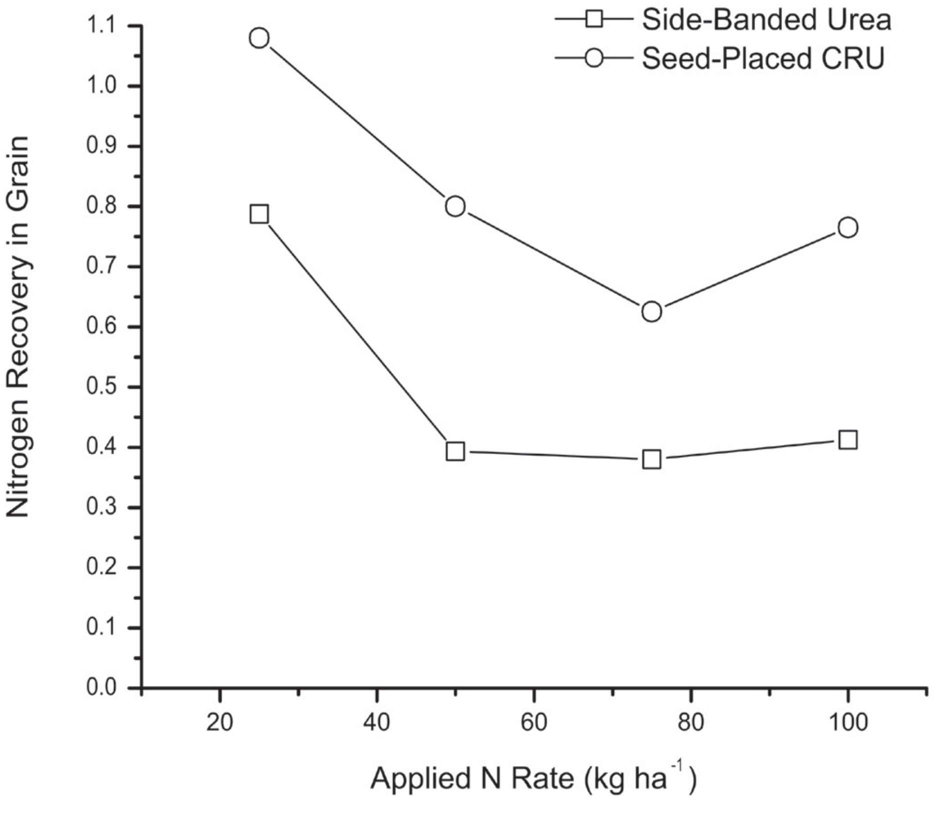

**FIGURE 5.** N recovery in grain from side-banded urea and seed-placed CRU treatments at Humboldt in 2000.

potential losses. Closer investigation of the site with the largest difference between treatments, Humboldt in 2000 (Fig. 5) indicates that at this site the mean difference in N use efficiency at the 100 kg N $ha^{-1}$ application level was 35%. This translates to an additional 26.4 kg N $ha^{-1}$ more removed from the seed-placed CRU treatment compared to the side-banded urea treatments at this site.

## CONCLUSIONS

Spring side-banded urea is accepted as an efficient N application method for wheat production in western Canada. We have demonstrated that seed-placed CRU is a viable alternative for N placement in this region. Seed-placed CRU produces similar yields and higher grain N content at equivalent rates compared to side-banded urea. Seed-row placement of CRU also eliminates the need for expensive side-banding equipment, or a separate fertilizer application pass, thus saving fuel and reducing the potential for soil erosion from more disturbance of the soil

Seed-placed CRU increases N fertilizer use efficiency. Average N recovery was increased by 4.2% across all sites, while one site had recovery increases as high as 35% compared to side-banded urea. The extent to which seed-placed CRU can increase N use efficiency is strongly linked to N application rate, N requirements of the crop, potential N loss through leaching and denitrification, and other yield-limiting factors, particularly soil moisture. A 4.2% improvement in N use efficiency across a large geographic region would translate into a substantial reduction in N loss from cropping using N fertilizer. Further, if we can identify situations where benefits from CRU are greatest, such as at Humboldt in 2000, the commercial acceptance of CRU could be quite widespread.

| | | |
|---|---|---|
| Received: | July | 18, 2001 |
| Revised: | October | 8, 2001 |
| Accepted: | October | 15, 2001 |
| Published: | October | 30, 2001 |
